# Ambiguous motivations in medical school applicants: a retrospective study from Japan

**DOI:** 10.1080/10872981.2025.2467487

**Published:** 2025-02-19

**Authors:** Asuka Kikuchi, Ryuichi Kawamoto, Masanori Abe, Daisuke Ninomiya, Yoshio Tokumoto, Teru Kumagi

**Affiliations:** a Department of General Practice, Ehime University Hospital, Toon City, Japan; b Department of Community Medicine, Ehime University, Toon City, Japan; c Center for General Clinical Training, Ehime University Hospital, Toon City, Japan

**Keywords:** Medical students, medical school applicants, motivation, intentions to become doctors, burnout, career immaturity, uncertain aspirations

## Abstract

Aspiring to become a physician is a natural expectation for applicants to medical school. However, choosing a career in medicine is a critical decision, especially in countries where high school students can apply to medical school without an undergraduate degree. Students may select a medical career for various reasons, including parental pressure and academic performance. The question of whether there are students who enroll in medical school without clear intentions of becoming doctors has not been extensively investigated in the literature. We conducted a retrospective study at a national university in Japan. Given the scarcity of research examining medical students who did not have a clear intention to become doctors at the time of admission, we created a survey. The questionnaire asked students whether they had clear intentions to become doctors upon enrollment, and those who responded affirmatively were defined as students with clear intentions to become doctors at admission and assigned to the first group. The second group was composed of students who entered medical school without clear intentions to become doctors. We then compared the differences in sociodemographic characteristics and career determinants between these groups using statistical methods, including Chi-square tests and logistic regression. The collection rate of the questionnaire was 76.2%. We found that 28.8% of students at a national medical school in Japan entered medical school without clear intentions of becoming doctors. For these students, ‘parental expectations’ and ‘peer influence’ were identified as significant career determinants. No sociodemographic characteristics showed significant associations with the ambiguity of students’ intentions to pursue a medical career at admission. This study confirmed that some students enroll in medical school without clear intentions of becoming doctors. The background factors related to this type of student were parental expectations and the influence of peers on career choice.

## Introduction

Choosing a medical career is an important decision for students. Medical school is difficult, the work of doctors is demanding, and medical school applicants are aware of these challenges [1–3]. In Japan, high school students can apply to medical schools without an undergraduate degree, which can lead to an even more difficult decision-making process, given the need for early career decisions [4]. However, even after being admitted to medical school, medical students and doctors face numerous hurdles, including maintaining motivation, staving off burnout, and facing the risk of academic failure, as documented in various studies [5–9].

Numerous studies have investigated the motivation of medical students from various perspectives. Research pertaining to the academic performance of medical students has indicated a positive correlation between high levels of intrinsic motivation and favorable grades as well as learning behaviors [10–12]. Research on curricula to enhance students’ motivation has also been actively conducted [13–15]. Additionally, studies exploring the psychological aspects of medical students have delved into topics such as stress and burnout experienced by medical students [11,16], as well as investigations into self-efficacy and altruism [17,18]. These studies collectively aim to elucidate the characteristics of medical students aspiring to become doctors, with the goal of determining how medical educators can better support their students. Such endeavors are crucial in facilitating the cultivation of competent healthcare professionals and ensuring the availability of sufficient human resources in the medical field [19,20].

Students’ motivations for entering medical school are varied and involve a complex interplay of internal and external factors, including personal ambition, societal expectations, reputation, and genuine desire to become a doctor [2,20–28]. Each factor can be either external or internal depending on the person [29]. Motivation consists of factors that are already established and non-modifiable at the time of admission, as well as factors that can be influenced by educators. Age, gender, personality traits, pre-existing social status, ethnicity and culture, support from teachers and parents, familial factors, and the academic year of the curriculum are examples of factors that are already present or determined at the time of admission to medical school and are therefore non-modifiable. On the other hand, factors such as autonomy, self-efficacy, admission procedures, and types of assessment can be influenced by educators. The interplay between non-modifiable factors and those that can be influenced, as well as their impact on intrinsic motivation, can lead to changes in the state of medical students’ motivation [29,30]. However, these factors only relate to the motivation for entering medical school and may not necessarily reflect a student’s aspirations to become a doctor.

Learning medicine is a very popular career path in Japan [4], and applicants are often high school students who may not have fully developed their career goals [31]. Even if they do not truly wish to enter medical school, it is undeniable that they may have chosen medical careers only because of their parents’ expectations, the influence of their siblings, or someone close [20,22,27,28], or they may have based their choice on good grades in high school [22]. Particularly, students who enter medical school based on family or cultural values are reported to be at higher risk of career indecision and long-term burnout after admission [16]. Considering these factors, it is essential for medical educators to appropriately assess and understand the career uncertainties and immaturity of medical students at the time of admission. However, to the best of the authors’ knowledge, there is no report that examines the existence of such medical students, namely, ‘students who enter medical school without clear intentions to become doctors.’ Understanding the prevalence and characteristics of these medical students is crucial for faculty members aiming to provide the necessary support to foster their development.

Therefore, this study seeks to shed light on the proportion of medical students with uncertain aspirations at admission and the factors influencing their educational and career trajectories.

## Method

### Objects

We conducted a retrospective observational study from April 2019 to March 2021 at Ehime University School of Medicine, a national university in Japan, using a self-administered questionnaire. All medical students attending the university were asked to complete the questionnaire during class or community practice. Students who were not obligated to attend these classes and sessions were invited via e-mail to answer the questionnaire online.

### The medical school entrance system in Japan

To clarify the study object, we will briefly introduce the Japanese medical education system. Medical schools in Japan do not require applicants to have undergraduate degrees. Therefore, most of the applicants are high school students. There are two types of medical schools, national and private. The tuition fee of national schools is only one tenth to one fifth of that of private medical schools. Therefore, the economic backgrounds of medical students at national universities range from poor to rich families. To apply to a national medical school, students must take the National Center Test for University Admission. Students who earn scores on the test over a certain level are then able to take the proprietary examinations that each national university provides and then attend interviews. However, some students are able to enter national medical schools upon recommendations from designated high schools; they are not required to take examinations but submit an essay and attend interviews. Of course, to obtain a recommendation, a student must have excellent grades during high school. Thus, regardless of the method of admission, all medical students at national universities have survived fierce competition. If students do not gain admission to a national medical school, private medical schools could be a second option. Because of their high tuition fees, these schools are available only those who can afford this option. Because the national center test for universities is not mandatory for private medical schools, some financially well-off students choose private schools without first enrolling in national medical schools [4,32].

Once students enter university, they are unable to change their major and must drop out and take another entrance examination the following year if they wish to change fields. This system forces students to take entrance examinations repeatedly, which can result in some students giving up on their career aspirations. Some people believe that spending years to get admitted to a university is related to personal problems or poor performance. This issue has led to discrimination against certain students during the admissions process [33]. Therefore, most medical school applicants begin to prepare for medical school admission early in their educations because they are aware of these hidden discriminatory features [4,32]. Retention rates are also a problem for medical students in Japan, with graduation rates reported to be 84.2%. The national average pass rate for the national medical examination has been 88.1% [34].

### Questionnaire

Given the paucity of research examining students who entered medical school without clear aspirations to become doctors, a proprietary questionnaire was devised to investigate this particular cohort. The questionnaire comprised three distinct sections: a primary section aimed at assessing the initial motivation to pursue a medical career upon enrollment, a sociodemographic background section, and a career determinants section. This approach follows the principles of mixed-methods research to effectively capture both quantitative data and qualitative narratives, which support theory building [35]. In designing the questionnaire categories, we primarily selected items that have been widely reported in previous research [29]. We focused on sociodemographic background factors and career determinants, while we chose not to include factors such as ambition, altruism, and the social reputation of being a physician from the default questionnaire items. This decision was made with consideration for the cultural tendencies of Japanese students, who often provide socially desirable responses when asked directly about personal traits, potentially obscuring their true motivations [36]. In contrast, sociodemographic background factors are objective and verifiable, as universities often have these records on file, derived from admission application documents. Students are aware of this, making it easier for them to respond honestly. Additionally, responses regarding career determinants at the time of admission do not affect students’ eligibility to enroll in medical school. Therefore, we determined that even students inclined to provide socially desirable responses would find these categories easier to answer. Below, we provide further details about the questionnaire.

In the primary section, we posed the question to students, ‘Did you have clear intentions to become a doctor at the time of admission?’ and instructed students to choose the option that is most closely aligned with their thoughts from the following options: ‘1. I had clear intentions to become a doctor. 2. I had considered other medical careers, and medical school was not mandatory. 3. Only because it was recommended by my parents (I did not originally intend to become a doctor). 4. Only because it was recommended by the cram school – a type of supplementary education school (I did not originally intend to become a doctor). 5. Only because I had a good score on the National Center Test for University Admission – Japan’s standardized university entrance exam (I did not originally intend to become a doctor). 6. Others.’ Students who chose option 6, ‘Others,’ were asked to provide a narrative account of their decision-making process. We defined students who chose option 1 as medical students with clear intentions to become doctors upon admission. We also investigated students’ original intended career paths.

The sociodemographic background section included the following items: sex, location and urbanization of the student’s hometown, type of high school, graduation from a combined junior and senior high school, experience of waiting more than a year for another chance to enter a university, a work experience program, withdrawal from a university to enter medical school, a scholarship, a recommendation from a designated high school, doctors in the students’ family, and doctors as role models. Certain sociodemographic characteristics were tailored to the Japanese higher education system [4].

In the career determinant factor section, we asked medical students what had the greatest impact on their career choice and instructed them to choose the option that best applied from the following options: parental expectations [20–22,27,28], the influence of siblings [20–22], the influence of high school classmates [22], or the illness of someone close [20]. In this section, similar to the previous section, we set a free-response section for students to provide explanations. We did not include the following options as career determinant factors: having scientific curiosity, helping others, and desiring the reputation of a doctor. All of these factors have been well reported in previous studies [2,20–28], and many subjects in this study used these keywords in the free-response sections. Therefore, we checked the free-response section, tabulated those key words, and then analyzed the data.

We added original survey items to the questionnaire based on a preliminary investigation conducted among 42 medical students from various grades, ranging from first-year to sixth-year. Students identified the popularity of work experience programs and admiration for medical TV shows and comic book characters as important motivational factors for high school students seeking admission to medical schools. The positive impact of work experience programs on potential medical school applicants has been suggested in prior research [37]; therefore, this factor was added as a determinant. Additionally, while there are limited reports on the influence of fictional doctors in medical TV shows and comic books on medical school applicants, existing evidence suggests their potential to enhance medical students’ motivation to learn [38,39]. Based on this, we also decided to include this factor as a determinant.

Sociodemographic characteristics were collected with Yes/No poll questions, and the factors with a great impact on students’ career choice at admission were collected with multiple-choice questions.

### Statistical analyses

Statistical analysis was performed using SPSS Statistics v27 (IBM Japan Digital Services Company). Questionnaires with missing data were excluded, and only those with complete data were used for analysis. First, the medical students were divided into two groups based on the results of the primary section: those with clear intentions to become doctors upon enrollment in medical school and those without. Next, Chi-square tests was conducted to examine whether there were differences in sociodemographic background factors and career determinant factors between these two groups. The dependent variables that showed an association with students without clear intentions to become doctors upon admission to medical school were determined. Logistic regression analysis was then conducted on the variables that exhibited significant differences in the Chi-square tests. Logistic regression analysis was conducted to adjust for potential confounding factors among variables that showed significant differences in the Chi-square test, ensuring the clarification of independent associations. Variables with *p* values < 0.05 in the Chi-square tests were included in the multivariate analysis. A *p* value < 0.05 was considered statistically significant.

### Informed consent and ethical considerations

The purpose of the survey was noted at the beginning of the questionnaire, and it was clearly explained that answering the questionnaire would constitute consent to cooperate in the study. We obtained the approval of the Institutional Review Board at Ehime University Hospital for this study (27 July 2015, Reference number; 15007004).

## Results

**Figure 1.**
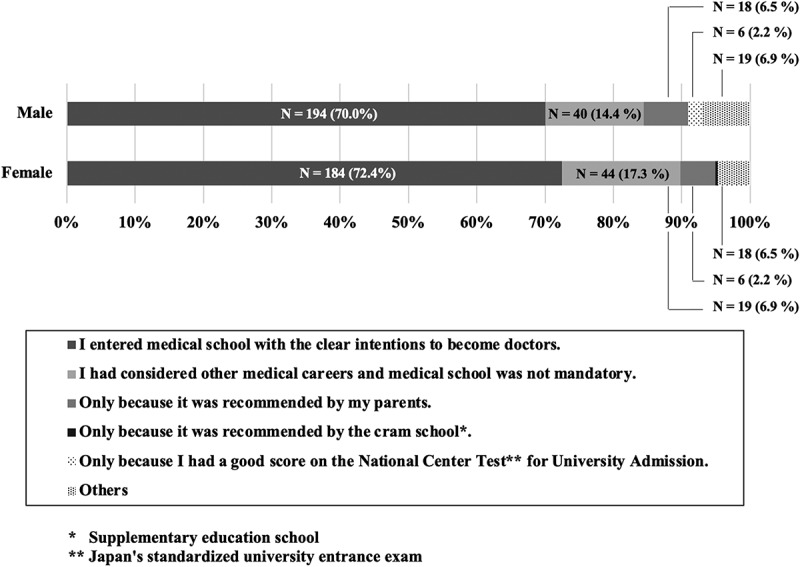
The reason why the students applied to medical school.

A total of 531 out of 697 students completed the questionnaire. The collection rate of the questionnaire was 76.2%, and none of the respondents withdrew from the study. A total of 153 (28.8%) students entered medical school without clear intentions to become doctors. No statistical significance was observed in the percentage of students without clear intentions to become doctors by gender (Figure 1). These students explained their reasons for choosing medical school as follows: ‘Considered other medical careers, and medical school was not mandatory’, ‘Only because it was recommended by my parents’, ‘Only because it was recommended by the cram school’, ‘Only because I had a good score on the National Centre Test for University Admission’, and other reasons. Other reasons included ‘Pure academic interest’, ‘Interpreted parents’ expectations (parents never mentioned their desire for their children to become doctors, but student sensed what their parents wanted)’, ‘For the reputation and career stability’, ‘I just chose what most of my classmates chose for their career’, ‘I just had good grades, so I chose to attend medical school’, and ‘For qualifications that will last a lifetime’ (Figure 1).

*Others included ‘pure academic interest,’ ‘interpreted parents’ expectations (parents never mentioned their desires that their children become doctors verbally, but I sensed what they wanted),’ ‘for the reputation and career stability,’ ‘I chose what most of my classmates chose for their career.’ ‘I just had good grades, so I chose to apply medical school.’ and ‘For qualifications that will last a lifetime.’

### Sociodemographic characteristics and career determinant factors behind students’ motivation at the time of admission

We next examined how the presence or absence of intentions to become a doctor upon admission to medical school was affected by sociodemographic characteristics and career-determining factors with Chi-square tests. Regarding sociodemographic characteristics, the students without clear intentions to become doctors have a significantly lower percentage of ‘the presence of doctors in students’ family members except for their parents’ and ‘the presence of doctor role models’ compared to those who do have intentions to become doctors (Table 1).

**Table 1.** Differences in sociodemographic characteristics between students with and without clear intentions to become doctors at admission.

Sociodemographic characteristics	I entered medical school with a clear intention to become a doctor. *N* (%)		
	Yes *N* = 378	No *N* = 153	*P*-value
Gender			
Male Female	194 (51.3%) 184 (48.7%)	83 (54.2%) 70 (45.8%)	0.541
Hometown is in the same prefecture where the medical school locates	180 (47.6%)	69 (45.1%)	0.598
Types of high school			
Public Non-public: Private, National, Others	183 (48.4%) 195 (51.6%)	66 (43.1%) 87 (56.9%)	0.270
Graduated from combined junior and senior high school	209 (55.3%)	86 (56.2%)	0.847
Having waited another chance to enter a university more than a year	161 (42.6%)	52 (34.0%)	0.067
A work experience program	15 (4.0%)	6 (3.9%)	0.980
Having withdrawn from a university and entered medical school	32 (8.5%)	8 (5.2%)	0.201
Scholarship	123 (32.5%)	48 (31.4%)	0.794
Recommendation by a designated school	117 (31.0%)	51 (33.3%)	0.593
The presence of doctors in students’ family member; parents	107 (28.3%)	39 (25.5%)	0.510
The presence of doctors in students’ family member except for their parents	117 (31.0%)	34 (22.2%)	**0.043**
Grown up in a rural area	99 (26.2%)	34 (22.2%)	0.339
The presence of doctor role-models	184 (48.7%)	35 (22.9%)	**<0.001**

Table 1 shows the difference of Sociodemographic characteristics between the students with and without clear intentions to become doctors at admission. *P*-values are from the Chi-square tests for categorical variables. Significant values (*p* < 0.05) are presented in bold.

In terms of factors that had a great impact on students’ career choices upon admission, the percentages of those citing ‘parental expectations’ and ‘peer influence’ were significantly higher among students who entered medical school without clear intentions to become doctors. Conversely, the percentages of those citing ‘the experience of someone’s illness’ and ‘the influence of medical TV dramas’ were significantly lower among such students (Table 2).

**Table 2.** The difference in factors that had a great impact on the decision making of career choice at admission between students with and without clear intentions to become doctors at admission.

Factors which had great impact on the decision making of students’ career choice at admission	I entered medical school with a clear intention to become a doctor. *N* (%)		
	Yes *N* = 378	No *N* = 153	*P*-value
Parental expectation	132 (34.9%)	79 (51.6%)	**<0.001**
The influence of one`s siblings	8 (2.1%)	8 (5.2%)	0.057
The influence of one’s peers	9 (2.4%)	12 (7.8%)	**0.003**
A workplace experience as an observing student	20 (5.3%)	10 (6.5%)	0.574
The experience of illness on someone close	91 (24.1%)	20 (13.1%)	**0.005**
The influence of medical TV dramas	38 (10.1%)	7 (4.6%)	**0.040**
The influence of medical comics	10 (2.6%)	6 (3.9%)	0.436
Scientific curiosity	24 (6.3%)	14 (9.2%)	0.257
Help others	58 (15.3%)	14 (9.2%)	0.059
Reputation	11 (2.9%)	4 (2.6%)	0.852

Table 2 shows the difference of factors which had great impact on the decision making of career choice at admission between the students with and without clear intentions to become doctors at admission. *P*-values are from the Chi-square tests for categorical variables. Significant values (*p* < 0.05) are presented in bold.

Finally, binomial logistic regression analysis was conducted to examine the factors associated with not having clear intentions to become doctors upon admission to medical school. The items that showed a significant association were ‘parental expectations’ (OR = 0.50, 95% CI: 0.32–0.79) and ‘influence of one’s peers on career decisions’ (OR = 0.35, 95% CI: 0.14–0.89), suggesting the possibility that group consensus and expectations within one’s social context may influence career choices (Table 3).

**Table 3.** Odds ratios of variables related to students with or without clear intentions to become doctors at admission.

Variables which related to clear intention to become doctor at the time of admission in Chi-square tests.	Students who entered medical school with clear intentions to become doctors. Yes = 378 No = 153			
	Stepwise regression		Forced regression	
	OR (95% CI)	*P*-value	OR (95% CI)	*P*-value
Sociodemographic characteristics				
The presence of doctors in students’ family member except for their parents	1.67 (1.04–2.68)	0.035	1.70 (1.06–2.74)	0.028
The presence of doctor role-models	3.21 (2.06–5.00)	<0.001	3.19 (2.04–4.99)	<0.001
Factors which had great impact on the decision making of students’ career choice at the time of admission.				
Parental expectation	**0.44 (0.29–0.66)**	**<0.001**	**0.50 (0.32–0.79)**	**0.003**
The influence of one’s peers on career decision	**0.32 (0.13–0.80)**	**0.015**	**0.35 (0.14–0.89)**	**0.028**
The experience of illness on someone close	–	–	1.62 (0.72–1.62)	0.110
The influence of medical TV dramas	2.08 (0.88–4.94)	0.098	2.56 (1.00–5.90)	0.049

Table 3 shows odds ratios (= OR) of variables which related to the students with or without clear intentions to become doctors at admission. Variables which showed significancy in the Chi-square tests were analyzed. Variables associated with the students without clear intention are showed in bold.

## Discussion

In this study, we found that 28.8% of students entered medical school without clear intentions to become doctors. We also found the associative factors of these students: ‘Parental expectations’ and ‘The influence of one’s peers’ on career decisions. To the authors’ knowledge, this is the first study to confirm the existence of students who enter medical school without a definitive intention to become doctors and to investigate their true motivations. This finding is consistent with patterns observed in other Asian contexts, where parental influence often plays a significant role in shaping career choices [18,40].

Several studies have reported a reduction in the motivational state of first-year medical students [41,42]. However, these studies did not explore the presence of medical students who did not have clear intentions to pursue a career in medicine upon admission. Additionally, while there are reports linking motivation decline and burnout among medical students [5–9,22,41,43], these studies did not clarify whether the burnout occurred after admission or if risk factors were already present upon entry to medical school. Among these studies, Goel et al. found that students with pre-existing depressive tendencies experienced worsening symptoms in their first year, and those who entered medical school due to parental expectations had higher depression scores and a greater risk of burnout [22]. These findings may align with our research, which identified students who entered medical school without a clear intention of becoming doctors.

In this study, we investigated the motivational factors of students without clear intentions to become doctors upon admission by utilizing both free-text and multiple-choice question formats. The most common reason for the application among such students was ‘I had considered other medical careers, and medical school was not mandatory’. This could be seen as ‘career immaturity’ because the students had chosen their career paths based on a vague notion of medical careers [31]. Given that even some medical students lack a concrete image of their future careers [44], it is unsurprising that high school students make ambiguous career choices. If high school students scored well on the National Center Test for University Admission and met the medical school application requirements, it is reasonable for students who originally intended to pursue medical careers to choose medicine. Further, the students who responded that ‘medical school was not mandatory’ were likely pursuing their ideal career paths in the medical profession, even if they did not have clear intentions to become doctors.

The second most common reason for medical school application among students without clear intentions was ‘Only because my parents recommended it’. This reason was also identified as a background factor in the numerical analysis, indicating a strong influence on their choice of career path. Several studies have reported the negative impact of ‘parental expectations’ as a career decision factor for medical students [30,32,40,45]. Among these studies, Griffin and Hu found that students who entered medical school due to parental expectations developed negative feelings toward their career paths as early as the end of their first year [30]. Their evaluations during the fifth year revealed an increased risk of long-term academic decline and burnout. While these negative perceptions were not specifically assessed at the point of admission, their findings suggest that parental expectations may contribute to early dissatisfaction and eventual burnout. This aligns with our results, which identified a similar association between entering medical school due to parental expectations and a lack of clear intentions to become doctors.

Additionally, when examining the influence of parental expectations on career choices at the time of university admission, it is important to consider cultural aspects, particularly for Asian students. Cooper et al. highlighted that Asian parents’ desire to spare their children from academic struggles and take pride in their success strongly influences career choices [45]. In Japan, this is further compounded by the practice of parents financing their children’s education [4,32,40]. In such situations, high school students are compelled to make career decisions based on their parents’ preferences. Consequently, their decision-making process is hindered, leading to the selection of a career path that aligns with their parents’ financial support. These cultural traits indicate that parental expectations serve as a potent motivating factor, highlighting a strong patriarchal intent, thereby restricting students’ autonomy in choosing their desired career paths. Such external factors strongly influence the decision-making process, which may explain the ambiguity that medical students experience in their desire to pursue a career in medicine [30].

However, it is important to be mindful of the negative effects of ‘parental expectations’ because in this survey, this was also the most common motivational factor for students who had clear intentions to become doctors. Although both groups were strongly influenced by parental expectations, their motivational states differed significantly in terms of clarity of their desire to become doctors. Understanding these differences is a challenge for future research. Clarifying them will deepen our understanding of medical students’ motivations and provide a foundation for supporting those who enter medical school without clear intentions. To address these challenges, subsequent research ought to investigate targeted interventions aimed at alleviating external pressures on medical students and explore how such interventions may help reduce the risk of burnout. Medical educators must carefully monitor medical students who enter with diverse motivations, paying attention to early signs of burnout and academic decline, and providing individualized support for career development that suits each student’s needs. Given the diverse motivations and external pressures faced by medical students, implementing structured support systems becomes essential. Previous research suggests that empathy- and motivation-driven curricula enhance student well-being and career commitment [15,17]. Implementing such approaches may ultimately serve as effective support strategies.

Finally, we discuss ‘the influence of one’s peers’ on career choices. In this study, ‘the influence of one’s peers’ was identified as an associative factor of medical students without clear intentions to become doctors. Previous research has shown that peer support and information-sharing can have positive effects on career decisions and promote career development [18,46]. However, the findings of this study indicate that peer influence did not strengthen students’ desire to become doctors; instead, it contributed to career uncertainty. In the qualitative research conducted alongside the quantitative evaluation, some students who lacked a clear intention to become doctors cited ‘because my peers chose medical school’ as their reason for applying, suggesting that peer influence plays a significant role as a background factor in ambiguous career choices. Such concordance in career choices among peers has been reported to stem from the tendency to readily accept peer opinions [47]. In Japan, high school students perceive college as a way to obtain an academic degree, social status, and a career, known as the ‘going on to higher education for now’ concept [48]. Considering these factors, it is possible that the influence of peers is one of the factors that students with immature career aspirations defer to in their career choices.

In addition, a study suggests that immature career aspirations among medical students may be linked to lower academic performance. She et al. divided medical students in their first to third years into three groups – those aspiring to become medical educators or researchers, clinicians, and those who were undecided – and evaluated their skill levels. Their findings indicated that students who were undecided about their career aspirations exhibited lower proficiency in essential skills such as experimental techniques, English proficiency, and computer literacy [44]. Although their study focused on post-graduation career planning, the uncertainty observed in these students resembles the cohort identified in our study. Therefore, students with immature career aspirations in our study may also be at risk of lower academic performance. This finding underscores the need for early interventions to support students with unclear career aspirations, to ensure they develop the essential skills required for academic success and professional fulfillment.

Medical students without clear intentions to become doctors may appear to be a homogeneous group characterized by immature career aspirations. However, career development is shaped by individual skills, attitudes, emotions, and life experiences. Therefore, even those who lack clear intentions may have diverse background factors, making them a heterogeneous group. Medical educators have a responsibility to recognize the varying motivational states of students and provide appropriate support. This is especially crucial when career decisions are made prematurely. By understanding these diverse motivations early on, educators can offer timely guidance and prevent potential misalignment between students’ goals and their career paths. To achieve this, medical schools should implement early screening tools and advisory programs to assist students with ambiguous career goals. Through these efforts, it is important for medical education to support students who initially lacked clear intentions to cultivate a concrete vision for their careers and successfully navigate their professional paths.

## Limitations of the study

This study has several limitations. First, the sample was racially homogenous, as it is uncommon for Japanese medical schools to admit students from diverse cultural backgrounds [4,40]. Consequently, the results may not be generalizable to medical students from other cultures with less patriarchal influence. Second, no existing questionnaire was found to investigate the research question regarding medical students’ intentions to become doctors at admission. Therefore, the questionnaire used in this study is original and has not undergone rigorous psychometric validation. While the mixed-methods approach applied in this study [35] effectively captures both quantitative data and qualitative narratives to support theory building, the reliance on self-reported data may have introduced response biases. This is particularly relevant given the cultural tendency of Japanese students to provide socially desirable answers [36], which might obscure their genuine motivations. Future studies should apply this questionnaire to further examine students’ career intentions and validate its reliability and usefulness. Third, the study was conducted between 2019 and 2021, coinciding with the COVID-19 pandemic. This period was marked by significant social and educational disruptions, which may have influenced students’ responses and career decision-making processes. The pandemic’s impact on the educational environment and students’ psychological state could have introduced biases or variations in the data. Therefore, these findings should be interpreted with caution, given the unique conditions during this timeframe.

## Conclusion

A survey of Japanese medical school entrants revealed that some students enter medical school without a clear desire to become doctors. Parental expectations and peer influence were identified as significant background factors affecting their career choices. This study underscores the necessity for medical schools to enhance their comprehension of the varied motivations of their students, especially in situations where career choices are made prematurely. Exploring specific interventions and support methods to alleviate external pressures, reduce the risk of burnout, and develop educational curricula that enhance students’ long-term commitment to their careers remains an important challenge for future research.

## Appendix

The questionnaire used in this study is shown below.

### Questionnaire Survey of Career Determinants of Medical Students

#### Purpose of the Questionnaire

This questionnaire is designed to determine the factors that influence students to choose medical school and careers. Our goal is to improve medical education as much as possible for our students. We would appreciate your cooperation.

### Questionnaire creator and purpose of use

This questionnaire was prepared by the Department of Community Medicine, Ehime University Graduate School of Medicine. By responding to the questionnaire, you consent to the use of your answers (statistical analysis and research use of the results). Please note that your answers to the questionnaire will not affect your academic performance in the Community Medicine course. **Although there is a question in the questionnaire regarding your true motivation for applying to universities, the content of your answers will not be passed on to the school administration. You are not obligated to answer questions that you do not wish to answer.**

## I. Please fill in the answer column with the appropriate answer about yourself

(A Sociodemographic section) **Q1**. Would you mind sharing your sex with me?

**Q2**. May I ask how old you are?

**Q3**. What grade are you in?

**Q4**. Where are you from?

**Q5**. Which type of high school did you attend?

1. Public 2. Private 3. National 4. Others

**Q6**. Was your high school a combined junior and senior high school?

1. Yes 2. No

**Q7**. Have you waited more than a year for another chance to enter a university?

1. Yes 2. No

**Q8**. Have you ever participated in a work experience program?

1. Yes 2. No

**Q9**. Did you drop out of a university and enroll in medical school?

1. Yes 2. No

**Q10**. Is your father or mother (or both parents) a physician?

1. Yes 2. No

**Q11**. Do you have a sibling/relative who is a physician?

1. Yes 2. No

**Q12**. Do you have a physician role model?

1. Yes 2. No

**Q13**. Do you receive funding for your studies from a local government or organization?

1. Yes 2. No

**Q14**. Did you enter a medical school with a recommendation?

1. Yes 2. No

**Q15**. What is the urban population of the area where you have lived the longest in your life?

1. Large city (over 500,000) 2. Medium-sized city (100,000 to 500,000)
2. Small city (approximately 50,000) 4. Town/village 5. Remote area/island

### II. We would like to ask you about your career choices when applying to college

**Q16–1**. Did you have a clear intention to become a doctor at the time of admission?

(A primary section)

1. I had a clear intention to become a doctor.
2. I had considered other medical careers, and medical school was not mandatory.
3. Only because it was recommended by my parents.

(I did not originally intend to become a doctor.)

4. Only because it was recommended by the cram school.

(I did not originally intend to become a doctor.)

5. Only because I had a good score on the National Center Test for University Admission. (I did not originally intend to become a doctor.)
6. Other than reasons 1 ~ 5

(In this case, please share your motivation for applying for medical school in 16–2.)

**Q16–2**. Please tell us what motivated you to apply to medical school and what is your strongest reason for applying.

**Q17**. If you chose 3 ~ 6 in Q16–1, please describe the career path you originally wanted to pursue.

**Q18**. Please select the factors that strongly influenced your decision to pursue a career. Please choose all applicable options. (Career determinant factor section.)

1. Parental expectations 2. The influence of my siblings
3. The influence of my peers 4. A workplace experience as an observing student
5. The influence of medical TV dramas 6. The influence of medical comics
7. The experience of illness of someone close. 8. Others:________
